# CRISPR-Cas13a system: A novel tool for molecular diagnostics

**DOI:** 10.3389/fmicb.2022.1060947

**Published:** 2022-12-08

**Authors:** Lixin Zhao, Minyue Qiu, Xiaojia Li, Juanzhen Yang, Jintao Li

**Affiliations:** ^1^Department of Biosafety, School of Basic Medicine, Army Medical University, Chongqing, China; ^2^Institute of Immunology, PLA, Army Medical University, Chongqing, China

**Keywords:** CRISPR-Cas, CRISPR/Cas13a, biosensor, SHERLOCK, molecular diagnostics

## Abstract

The clustered regularly interspaced short palindromic repeats (CRISPR) system is a natural adaptive immune system of prokaryotes. The CRISPR-Cas system is currently divided into two classes and six types: types I, III, and IV in class 1 systems and types II, V, and VI in class 2 systems. Among the CRISPR-Cas type VI systems, the CRISPR/Cas13a system has been the most widely characterized for its application in molecular diagnostics, gene therapy, gene editing, and RNA imaging. Moreover, because of the trans-cleavage activity of Cas13a and the high specificity of its CRISPR RNA, the CRISPR/Cas13a system has enormous potential in the field of molecular diagnostics. Herein, we summarize the applications of the CRISPR/Cas13a system in the detection of pathogens, including viruses, bacteria, parasites, chlamydia, and fungus; biomarkers, such as microRNAs, lncRNAs, and circRNAs; and some non-nucleic acid targets, including proteins, ions, and methyl groups. Meanwhile, we highlight the working principles of some novel Cas13a-based detection methods, including the Specific High-Sensitivity Enzymatic Reporter UnLOCKing (SHERLOCK) and its improved versions, Cas13a-based nucleic acid amplification-free biosensors, and Cas13a-based biosensors for non-nucleic acid target detection. Finally, we focus on some issues that need to be solved and the development prospects of the CRISPR/Cas13a system.

## Introduction

The clustered regularly interspaced short palindromic repeats (CRISPR) system, which exists in almost half of bacteria (40%) and almost all archaea (90%) (Grissa et al., 2007; Kunin et al., 2007), is the natural adaptive immune system for prokaryotes to defend against extraneous invasion by phages and transformation of plasmids (Sorek et al., 2008; Garneau et al., 2010). The CRISPR array, which comprises short direct repeats separated by spacer sequences acquired from foreign nucleic acids (Mojica et al., 2005; Hochstrasser and Doudna, 2015), was first discovered in 1987 (Ishino et al., 1987). Additional elements of the CRISPR system include the leader sequence and CRISPR-associated (Cas) genes (Pougach et al., 2010; Yosef et al., 2012; Makarova et al., 2015). The mechanism of adaptive immunity driven by the CRISPR system includes three common stages (van der Oost et al., 2014; Amitai and Sorek, 2016; Sternberg et al., 2016; Hille et al., 2018; Wang et al., 2019): (1) the adaption stage, in which the CRISPR system recognizes foreign nucleic acids and integrates them into spacer sequences in the CRISPR array to form immunologic memory; (2) the CRISPR-RNA (crRNA) expression and maturation stage, in which the CRISPR array is transcribed into precursor CRISPR-RNA (pre-crRNA) that is subsequently processed to mature crRNA; (3) the interference stage, in which Cas protein–crRNA complex targets and cleaves the identified foreign DNAs and RNAs (Marraffini and Sontheimer, 2008; Hale et al., 2009).

**Figure 1:**
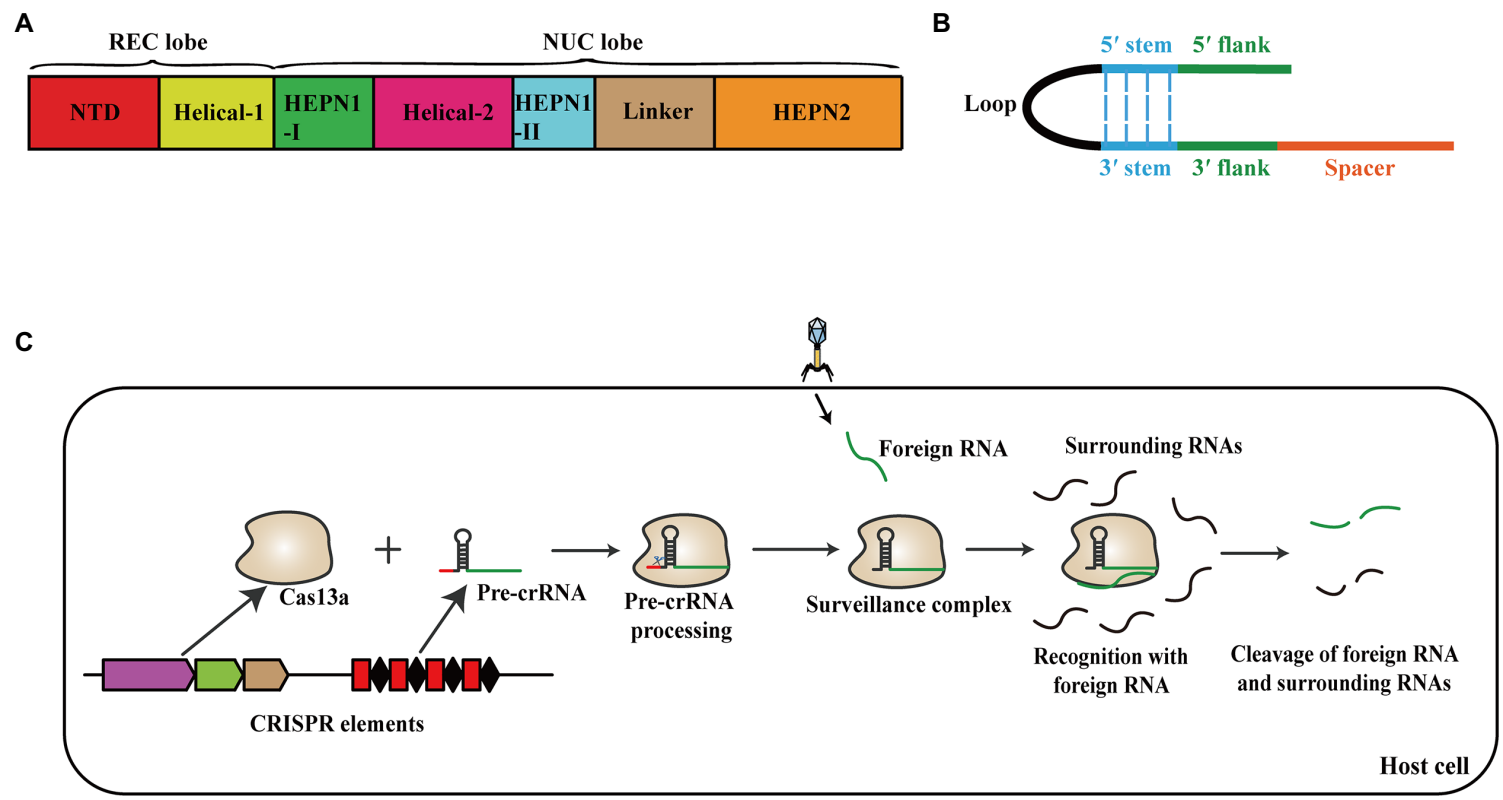
**(A)** Domain organization schematic of Cas13a. Cas13a comprises REC lobe and NUC lobe. The Helical-1 domain and the N-terminal domain constitute REC lobe, the NUC lobe contains the HEPN1 domain, HEPN2 domain, Helical-2 domain, and a Linker. **(B)** Schematic representation of the mature crRNA structure. The mature crRNA contains 3′ spacer and 5′ handle, which consists of 5′ flank, 5′ stem, Loop, 3′ stem, and 3′ flank. **(C)** Schematic of the effect mechanism of Cas13a system against foreign nucleic acid. Cas13a processes pre-crRNA into mature crRNA independently and combines with its crRNA to form the surveillance complex that recognizes the foreign target RNA and cleaves the target and surrounding RNAs.

The currently discovered CRISPR-Cas system is generally divided into two classes, six types, and 33 subtypes after multiple refinements (Makarova et al., 2011, 2015, 2020). The class 1 systems, including types I, III, and IV, are represented by multi-protein effecter complexes, while the class 2 systems, containing types II, V, and VI, utilize a single, large, multi-domain Cas protein as the effecter module (Mohanraju et al., 2016; Koonin et al., 2017). The CRISPR VI systems have four subtypes: VI-A, VI-B, VI-C, and VI-D, which are also termed Cas13a, Cas13b, Cas13c, and Cas13d systems, respectively (Makarova et al., 2020). Cas13a (also known as C2c2) in type VI systems is an RNA-guided RNase, which contains two conserved HEPN (Higher Eukaryotes and Prokaryotes Nucleotide-binding) domains (Abudayyeh et al., 2016). Cas13a comprises two lobes termed the crRNA-recognition (REC) lobe and the nuclease (NUC) lobe. The Helical-1 domain and the N-terminal domain (NTD) constitute the REC lobe, whereas the NUC lobe contains the HEPN1 domain, HEPN2 domain, Helical-2 domain, and a Linker between two HEPN domains (Figure 1A; Liu et al., 2017). The mature crRNA contains a 3′ spacer and a 5′ handle, which is divided into 5′ flank, 5′ stem, Loop, 3′ stem, and 3′ flank (Figure 1B; Knott et al., 2017). Cas13a processes pre-crRNA into mature crRNA independently and combines with its crRNA to form the surveillance complex that recognizes the foreign target RNA and cleaves the target and surrounding RNAs (Figure 1C; Knott et al., 2017).

Cas13a possesses two RNase activities, one for pre-crRNA processing and the other for cis-cleavage of the target RNA and trans-cleavage of non-specific RNAs. The pre-crRNA is cleaved in a metal-independent and acid–base manner by conserved residues in the Helical-1 and HEPN2 domains (Knott et al., 2017). The position of processing generally occurs 2–5 nucleotides upstream of the repeat sequences of crRNA (East-Seletsky et al., 2016). The two nucleotides adjacent to the broken site are essential for processing activity. Notably, the processing of pre-crRNA is not necessary for target cleavage because effecter complexes with pre-crRNA still have cleavage capacity (East-Seletsky et al., 2017). In addition to the common target RNA cleavage mechanism (cis-cleavage), Cas13a also exhibits non-specific collateral cleavage activity of the surrounding non-target RNAs; this collateral activity can achieve robust signal amplification with 10^4^ turnover ability (East-Seletsky et al., 2016). The cis- and trans-cleavage activities of Cas13a are dominated by two HEPN domains in a divalent cation-dependent manner (Nalefski et al., 2021). Different Cas13a orthologs exhibit A or U preferences for the cleavage site (East-Seletsky et al., 2017). The length of the spacer and the number of and position of mismatches between the spacer and target RNA are all vital for activating the RNase activity of Cas13a. The spacer length is required to be as short as 20 nt to retain the cleavage ability of Cas13a (Abudayyeh et al., 2017). A single mismatch between the crRNA and the target has a minor effect on the cleavage activity of Cas13a, and double mismatches greatly reduce the cleavage activity. Meanwhile, the “central seed region” of spacer-target duplex is more sensitive to mismatches (Abudayyeh et al., 2016). Intriguingly, Tambe et al. have demonstrated that the target binding affinity of Cas13a was uncoupled with the activation of RNase activity in LbuCas13a, and they found a “HEPN-nuclease switch region,” in which mismatches result in unaltered target binding affinity but lead to inactivation of the HEPN-nuclease (Tambe et al., 2018).

**Figure 2:**
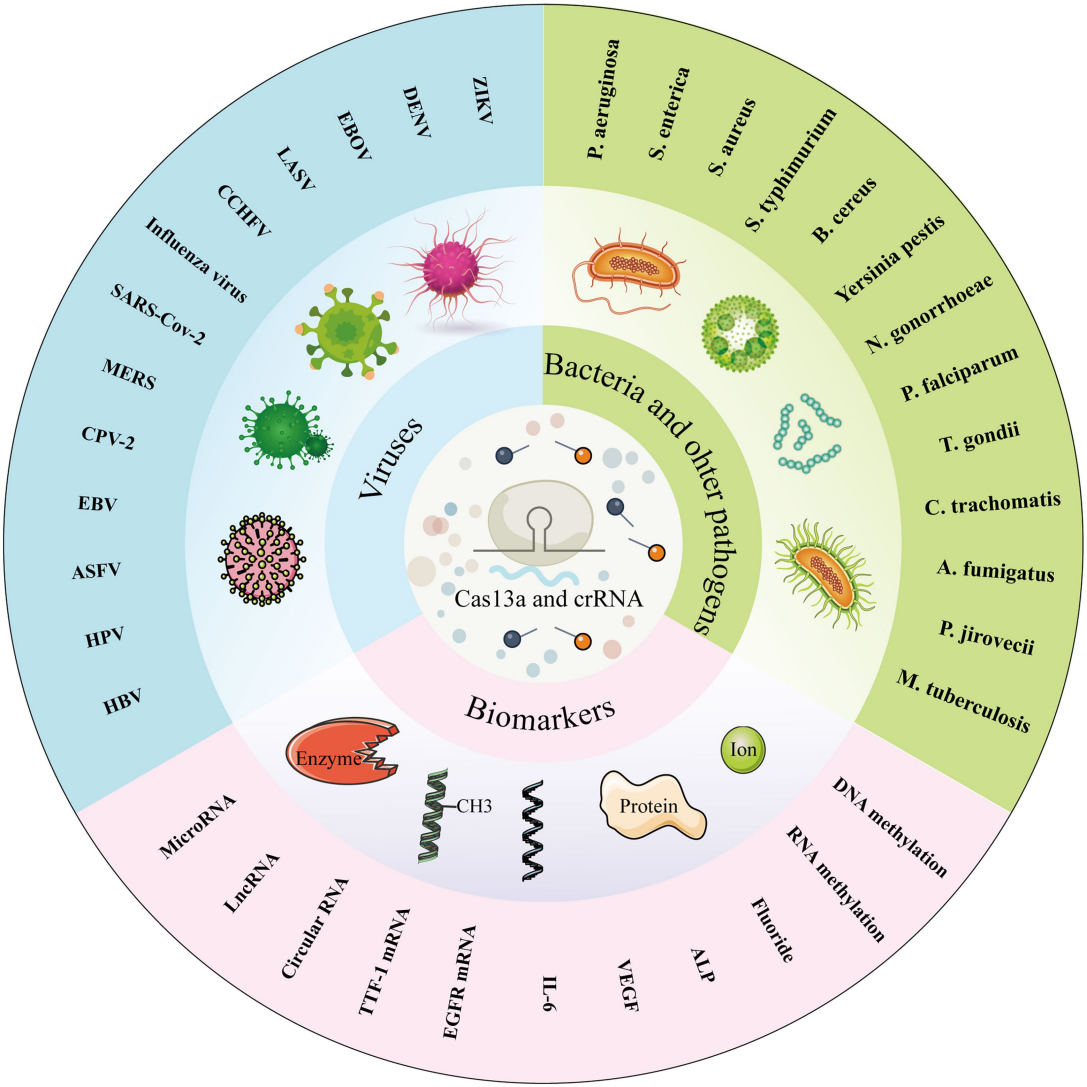
Various targets detected by Cas13a-based biosensors. Cas13a-based biosensors are able to detect pathogens, including viruses, bacteria, parasites, chlamydia, and fungus; biomarkers, such as microRNAs, IncRNAs, and circRNAs; and some non-nucleic acid targets, including proteins, ions, and methyl groups.

As the most widely characterized system of CRISPR, Cas13a system has been applied for molecular diagnostics, gene therapy, gene editing, and RNA imaging (Abudayyeh et al., 2017; Cox et al., 2017; Gootenberg et al., 2017; Li et al., 2020). Among the numerous applications of the Cas13a system, we focused on its application in molecular diagnostics since various studies have been reported in this field and Cas13a-based biosensors have made great progress. The activated collateral cleavage activity of Cas13a and the simple programmability of crRNA provide tremendous potential for Cas13a-based molecular diagnostics. East et al. first detected the bacteriophage λ gene by harnessing the RNA-guided trans-cleavage activity of LbuCas13a, in which activated LbuCas13a, followed by specific crRNA-target recognition, can cleave fluorophore quencher-labeled reporter RNA to cause increased fluorescence (East-Seletsky et al., 2016). Since then, CRISPR/Cas13a-based biosensors have become the spotlight in the field of molecular diagnostics and have been applied for the detection of various kinds of targets. The CRISPR/Cas13a system has been developed as an effective tool for molecular diagnostics, with expectations to improve the sensitivity, specificity, operability, portability and cost performance of molecular diagnostics tools (such as antigen–antibody reaction, PCR-based techniques, genome sequencing, etc.). In this review, we summarize the previously reported applications of the CRISPR/Cas13a system in molecular diagnostics for detecting pathogens, including viruses, bacteria, parasites, chlamydia, and fungus; biomarkers, such as microRNAs, lncRNAs, and circRNAs; proteins; ions; and methyl groups (Figure 2). The specific detection methods, targets, amplification methods, readout, sensitivity, and running time-based CRISPR/Cas13a detection technology are listed in Table 1. We also detail the working principles of some CRISPR/Cas13a-based detection methods, including the Specific High-Sensitivity Enzymatic Reporter UnLOCKing (SHERLOCK) and its improved versions, Cas13a-based nucleic acid amplification-free biosensors, and Cas13a-based biosensors for non-nucleic acid target detection. Finally, we point out some issues associated with CRISPR/Cas13a-based biosensors that need to be solved and focus on the future development of Cas13a-based biosensors.

**Table 1:** CRISPR/Cas13a-based biosensors for various targets.

Detection method	Target	Amplification method	Readout	LOD	Time	References
SHERLOCK	ZIKV, DENV	RT-RPA	Fluorescence	2 aM	>3 h	Gootenberg et al. (2017)
SHERLOCKv2	ZIKV, DENV	RT-RPA	Fluorescence, Lateral flow	2 aM (Lateral flow)	<90 min	Gootenberg et al. (2018)
SHERLOCK coupled with HUDSON	ZIKV, DENV	RT-RPA	Fluorescence, Lateral flow	0.9 aM (Fluorescence)	<2 h	Myhrvold et al. (2018)
Cas13a-based electrochemical biosensor coupled with CHA amplification	DENV-1	CHA	Electrochemistry	0.78 fM	>90 min	Wang J. et al. (2021)
Cas13a-based biosensor coupled with microfluidic system	EBOV	None	Fluorescence	5. 45 × 10^4^ copies/μl	>25 min	Qin et al. (2019)
SHERLOCK coupled with HUDSON	EBOV, LASV	RT-RPA	Fluorescence, Lateral flow	10 copies/μl (Fluorescence) 100 copies/μl (Lateral flow)	>2 h (Fluorescence)	Barnes et al. (2020)
Cas-Roller	EBOV	DNA roller	Fluorescence	291 aM	~40 min	Hang et al. (2022)
RPA-SHERLOCK	CCHFV	RT-RPA	Fluorescence	1 copy/μl	30–40 min	Li et al. (2022a)
RPA-SHERLOCK	Avian Influenza A H7N9 Virus	RT-RPA	Fluorescence	1 fM	50 min	Liu et al. (2019)
RPA-SHERLOCK	SARS-CoV-2	RT-RPA	Fluorescence, Lateral flow	42 copies per reaction	70 min	Patchsung et al. (2020)
Cas13a-based test strip coupled with quantum dot microspheres	SARS-CoV-2	RT-RAA	Lateral flow	1 copy/ml	1 h	Zhang Q. et al. (2022)
RT-LAMP-SHERLOCK	SARS-CoV-2	RT-LAMP	Fluorescence	6.75 copies/μl	50 min	Khan et al. (2021)
SHINE	SARS-CoV-2	RT-RPA	Fluorescence, Lateral flow	10 copies/μl (Fluorescence) 100 copies/μl (Lateral flow)	50 min	Arizti-Sanz et al. (2020)
CASSPIT	SARS-CoV-2	RT-RPA	Lateral flow	200 copies per reaction	>50 min	Azmi et al. (2021)
RT-LAMP-CRISPR-Cas13a	SARS-CoV-2	RT-LAMP	Lateral flow	10 copies	<2 h	Ortiz-Cartagena et al. (2022)
Cas13a-based biosensor using combination of crRNAs	SARS-CoV-2	None	Fluorescence	31 copies/μl	30 min	Fozouni et al. (2021)
Ultralocalized Cas13a assay	SARS-CoV-2, P. aeruginosa, and MiR-17	None	Fluorescence	6 copies/μl (SARS-CoV-2) 6 × 10^3^ copies/μl (P. Aeruginosa) 10 fM (MiR-17)	60 min	Tian et al. (2021)
opn-SATORI	SARS-CoV-2	None	Fluorescence	3.9 copies/μl	9 min	Shinoda et al. (2022)
Cas13a-based electrochemical biosensor	SARS-CoV-2	None	Electrochemistry	4.4 × 10^−2^ fg/ml (ORF gene) 8.1 × 10^−2^ fg/ml (S gene)	~1.5 h	Heo et al. (2022)
Cas13a-gFET	SARS-CoV-2	None	Electrochemistry	1 aM	30 min	Li et al. (2022b)
CASCADE	SARS-CoV-2	RT-RPA, NASBA	Colorimetry	3 fM (RPA) 40 aM (NASBA)	>2 h (RPA) 2 h (NASBA)	López-Valls et al. (2022)
PCR-CRISPR	SARS-CoV-2	RT-PCR	Fluorescence	1 copy/μl	>70 min	Niu et al. (2022)
Light-up CRISPR-Cas13 transcription amplification method	MERS, SARS, SARS-CoV-2, and Influenza Virus	Transcription amplification, light-up RNA aptamer	Fluorescence	82 copies per reaction (SARS-CoV-2)	>90 min	Wang Y. et al. (2021)
Cas13C	SARS-CoV-2	NASBA	Fluorescence	0.216 fM per reaction	>2.5 h	Wang et al. (2022)
Cas13a-based biosensor coupled with hybridization chain reaction amplification	SARS-CoV-2	Hybridization chain reaction (HCR)	Fluorescence	10 aM (ORF gene, S gene) 100 aM (N gene)	1 h	Yang et al. (2021)
RPA-SHERLOCK	CPV-2	RPA	Fluorescence	100 amol/L	30 min	Khan et al. (2019)
Room temperature SHERLOCK	EBV	RPA	Fluorescence	8 copies	——	Wu et al. (2019)
SHERLOCK	ASFV	RAA	Lateral flow	10 copies/μl	1 h	Wei et al. (2022)
One pot SHERLOCK	ASFV	RPA	Fluorescence	3 copies/μl	25 min	Hu et al. (2022)
PADLOCK	HPV-16	Droplet digital RPA (ddRPA)	Fluorescence	5 copies/μl	30 min	Cui et al. (2022)
RCA-PCR-CRIPSR	HBV	RCA, PCR	Fluorescence	1 copy/μl	>1 d	Zhang X. et al. (2022)
One Tube SHERLOCK	Salmonella spp.	RPA	Fluorescence	10^2^ copies/μl	20 min	An et al. (2021)
APC-Cas	S. enteritidis	Allosteric probe-initiated catalysis	Fluorescence	1 CFU/ml	140 min	Shen et al. (2020)
CRISPR/Cas13-based NASBA assay	S. enterica	NASBA	Fluorescence	1.5 CFU/ml	<2.5 h	Xue et al. (2022)
PCR-Cas13a	S. aureus	PCR	Fluorescence	1 CFU/ml	<4 h	Zhou J. et al. (2020)
PCR-Cas13a	S. typhimurium	PCR	Fluorescence	1 CFU/ml	2 h	Gao et al. (2021)
Light-up CRISPR-Cas13 transcription amplification method	B. cereus	Light-up RNA aptamer	Fluorescence	10 CFU/ml	>20 min	Zhang et al. (2021)
RPA-SHERLOCK	Yersinia pestis	RPA	Fluorescence	420 copies/ml	~4 h	Schultzhaus et al. (2021)
RPA-SHERLOCK	Neisseria gonorrhoeae	RPA	Fluorescence	10 copies/μl	>5 h	Luo et al. (2021)
RPA-SHERLOCK	P. falciparum	RPA	Fluorescence	4.2 aM	> 3.5 h	Cunningham et al. (2021)
Cas13a-based biosensor coupled with RAA	T. gondii	RAA	Lateral flow	1 × 10^−6^ ng/μl	2 h	Zhao et al. (2022)
RPA-SHERLOCK	C. trachomatis	RPA	Fluorescence	10 fM	2 h	Huang et al. (2021)
RPA-SHERLOCK	Aspergillus fumigatus	RPA	Fluorescence	3 copies/L	70 min	Li et al. (2021)
Cas13a-based biosensor coupled with TMA	Pneumocystis jirovecii	TMA	Fluorescence	2 copies/μl	>80 min	Zhan et al. (2022)
PCR-Cas13a	Mycobacterium tuberculosis	PCR	Fluorescence	1 copy/μl (S91P) to 100 copies/μl (D94G)	> 12 h	Bai et al. (2022)
Cas13a reaction	MiR-17	None	Fluorescence	4.5 amol	30 min	Shan et al. (2019)
Cas13a-based biosensor coupled with gold nanoparticles-based colorimetric assay	MiR-17	None	Colorimetry	500 fM	~20 min	Yuan et al. (2020)
PECL-CRISPR	MiR-17	Isothermal exponential amplification (EXPAR)	Electrochemiluminescence	1 fM	>100 min	Zhou T. et al. (2020)
casCRISPR	MiR-17	None	Fluorescence	1.33 fM	100 min	Sha et al. (2021)
Cas13a-based electrochemical biosensor	MiR19b, MiR20a	None	Electrochemistry	10 pM	<4 h	Bruch et al. (2019)
Cas13a-based electrochemical biosensor	MiR-21	CHA	Electrochemistry	2.6 fM	>2 h	Cui et al. (2021)
Cas13a-based biosensor coupled with magnetic relaxation switching	MiR-21	None	T2 signal	0.22 pM	60 min	Guo et al. (2022)
vCas	MiR-10b	RCA	Colorimetry	1 fM	~2 h	Zhou et al. (2021)
p-FLISA	LncRNA H19	None	Fluorescence	6 pM	>3,5 h	Liu W. J. et al. (2021)
Cas13a induced exponential amplification assay	ciRS-7	LAMP	Fluorescence	1 fM	30 min	Song et al. (2022)
CRISPR-Biosensor X	MiR19b, MiR20a	None	Electrochemistry	2 pM	~3.5 h	Bruch et al. (2021)
COMET	MiR-17, MiR-155, MiR-19b, MiR-210, TTF-1 mRNA, and EGFR mRNA	CHDC	Electrochemistry	50 aM	36 min	Sheng et al. (2021)
CLISA	IL-6, VEGF	None	Fluorescence	2. 29 fM (IL-6) 0.81 fM (VEGF)	>5 h	Chen et al. (2020)
TITAC-Cas	ALP	RPA	Fluorescence	6 ± 0. 52 mU/L	>85 min	Wang et al. (2021a)
Cas13a-based biosensor coupled with fluoride riboswitch-regulated transcription	Fluoride	None	Fluorescence	1.7 uM	30 min	Ma et al. (2021)
SPRINT	Cofactors, Nucleotides, Metabolites of amino acids, Tetracycline, and Monatomic ions	None	Fluorescence	——	——	Iwasaki and Batey (2020)
Cas13a reaction	N1-methyladenosine (m1A)	None	Fluorescence	——	25 min	Chen et al. (2019)
Cas13a reaction	N6-methyladenosine (m6A)	None	Fluorescence	——	25 min	Yang et al. (2020)
DESCS	DNA C5 methylation	None	Fluorescence, Lateral flow	86. 4 aM (Fluorescence) 200 aM (Lateral flow)	>2 h	Wang et al. (2021b)

## Cas13a-based detection for viruses

### Flaviviruses

**Figure 3:**
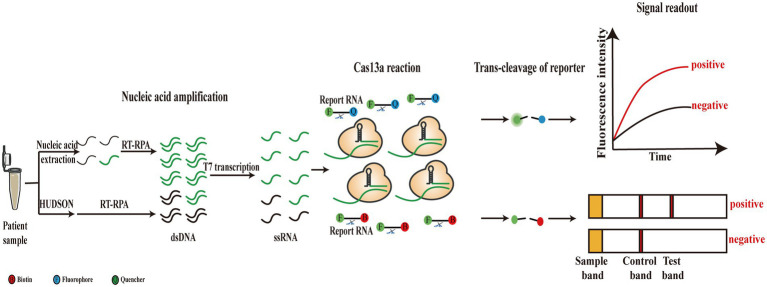
Schematic of original SHERLOCK and its improved versions. Target RNAs are extracted from patient sample and then amplified through RT-RPA and T7 transcription. Subsequently, amplification products are recognized and trans-cleaved by Cas13a system with fluorescence or lateral flow readout. Patient sample can also be directly treated by HUDSON method without the extraction step.

The global pandemic of flaviviruses, including Dengue virus (DENV), Zika virus (ZIKV), West Nile virus (WNV), and Yellow fever virus (YFV), has placed a huge burden on global health for the last several decades. Among these viruses, DENV causes nearly 400 million infections each year, and the Dengue fever epidemic has affected a quarter of the world’s population (Pierson and Diamond, 2020). In addition, the spread of ZIKV in recent years also led to numerous infection cases in the American region (Pierson and Diamond, 2018). The serious problems caused by flaviviruses have made rapid and accurate diagnosis of the viruses essential. A novel CRISPR-based nucleic acid detection platform has been established, called the SHERLOCK, which combines isothermal amplification that comprises recombinase polymerase amplification (RPA) and T7 transcription with the collateral cleavage effect of Cas13a for virus detection (Figure 3). Because of the isothermal amplification of the target RNA and the specificity of crRNA-guided recognition, the SHERLOCK assay achieved attomolar sensitivity and single-base specificity for ZIKV and DENV detection. Furthermore, SHERLOCK is also available in paper spotting and lyophilization forms, which significantly improve its flexibility and portability (Gootenberg et al., 2017; Kellner et al., 2019). Before long, Gootenberg et al. developed the SHERLOCK version 2 (SHERLOCKv2) by refining the original method. This method combined CRISPR/Cas13a-based detection with four multiplexing channels and achieved instrument-free detection of DENV or ZIKV ssRNA within 90 min, with a sensitivity of 2 aM. In addition to fluorescence readout, the lateral-flow test was employed for SHERLOCKv2 as a visual readout for field applications (Figure 3). Furthermore, the use of Csm6, an auxiliary CRISPR-associated enzyme, significantly increased the sensitivity of RPA-SHERLOCK (Gootenberg et al., 2018). In general, field-deployable tests can detect clinical samples without extracting nucleic acids. Therefore, an extraction-free method was reported, called heating unextracted diagnostic samples to obliterate nucleases (HUDSON), in which samples are directly added to the RPA process after HUDSON (37°C–50°C, 5–20 min for nuclease inactivation and 64°C–95°C, 5 min for viral inactivation) treatment without purification or dilution (Figure 3). The HUDSON method combined with RPA-SHERLOCK was used to directly detect DENV in patient samples within 2 h, and four serotypes of DENV were distinguished using this method. ZIKV and viral single-nucleotide polymorphisms (SNP) were also confirmed. Furthermore, this extraction-free assay still achieved attomolar detection sensitivity (Myhrvold et al., 2018). Recently, one research reported a novel electrochemical method in which the Cas13a system was combined with catalytic hairpin assembly (CHA) signal amplification. In this method, the activated Cas13a cleaves the reporter RNA hybridized to the swing arm-DNA walker (DW), promoting a series of downstream strand displacement reactions to produce electrochemical signals. Using this assay, they could detect DENV-1 at a level as low as 0.78 fM (Wang J. et al., 2021).

### Hemorrhagic fever viruses

The Ebola virus (EBOV) is a member of the Filoviridae and can result in the fulminating infectious disease, Ebola hemorrhagic fever, which is the deadliest viral hemorrhagic fever. The outbreak of Ebola in 2014 resulted in over 28,000 infected cases and over 11,000 deaths (Cenciarelli et al., 2015). Similarly, the Lassa virus (LASV), a single-stranded negative-strand RNA virus of the family Arenavirus, generally causes severe Lassa fever, which leads to approximately 5,000–10,000 deaths per year (Happi et al., 2019). Both the two hemorrhagic fever viruses constitute serious global health threats. However, the diagnosis of these two diseases is challenging owing to non-specific symptoms. Therefore, sensitive and specific methods for point-of-care detection of the Ebola and Lassa virus are required. Qin et al. reported an automated microfluidic system combined with the collateral activity of Cas13a for the detection of EBOV. Using a sensitive custom integrated fluorometer for reading fluorescence signals, they achieved a limit of detection (LOD) of 5. 45 × 10^4^ copies/μl for EBOV oligos without target RNA amplification and improved point-of-care testing for the EBOV (Qin et al., 2019). Moreover, the previously reported HUDSON method combined with RPA-SHERLOCK was employed to detect the Ebola and Lassa viruses, with an LOD of as low as 10 copies/μl, in which the fluorescent readout of LASV-II was 100% consistent with the results of reverse-transcriptase quantitative polymerase chain reaction (RT-qPCR). They also detected EBOV in the whole blood, urine, and saliva samples using this approach (Barnes et al., 2020). Recently, a novel method called the Cas-Roller was reported, which combines the Cas13a reaction with a DNA roller reaction to detect Ebola RNA and achieved an LOD of 291 aM within 40 min (Hang et al., 2022). In addition, the RPA-SHERLOCK method was employed to detect the Crimean-Congo hemorrhagic fever virus (CCHFV), another severe hemorrhagic fever virus (Bente et al., 2013), realizing an LOD of 1 copy/μl within 30–40 min and discriminating CCHFV from a series of CCHFV-related viruses (Li et al., 2022a).

### Respiratory viruses

Influenza viruses have spread worldwide for decades, and each outbreak has caused severe destruction to human society. In 2013, a new influenza virus H7N9 was found in China; since then, 1,568 laboratory-confirmed H7N9 infected cases have been reported with the mortality rate up to 39.2% (Gao et al., 2013; Liu W. J. et al., 2021). Therefore, establishing a sensitive and rapid method for the detection of the H7N9 virus is required. A previous study applied Cas13-based detection for H7N9 virus *via* targeting its hemagglutinin (HA) and neuraminidase (NA) genes. They could detect 1 nM of NA ssRNA within 5 min without amplification, and the method could test 1 fM of HA ssRNA in 50 min when combined with the RPA-SHERLOCK assay (Liu et al., 2019).

**Figure 4:**
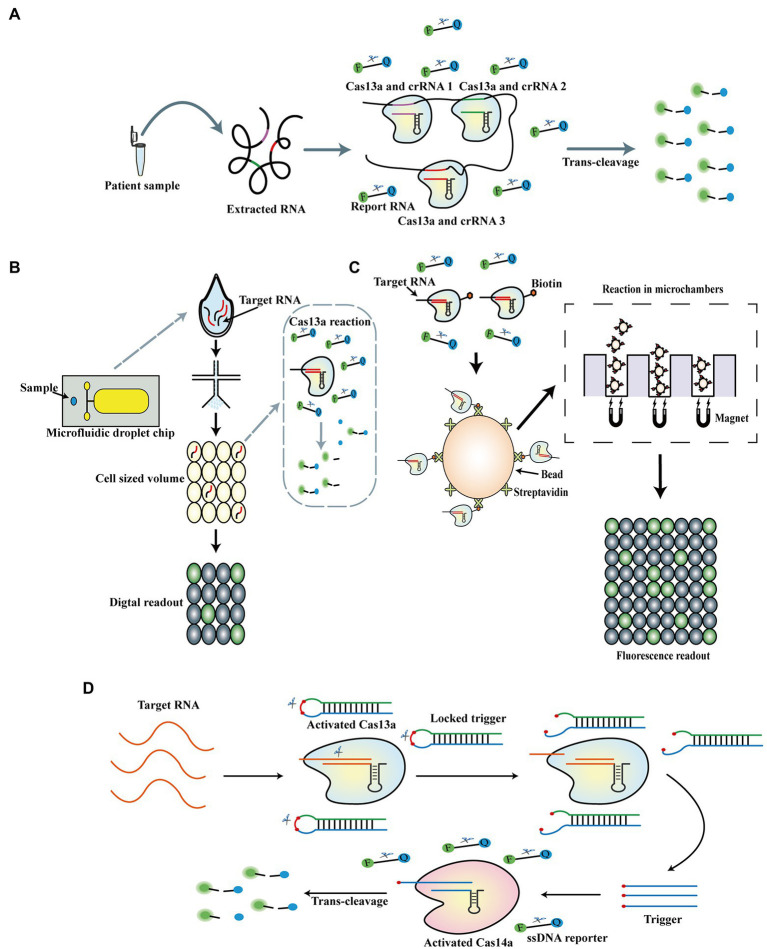
Cas13a-based nucleic acid amplification-free biosensors. **(A)** Schematic of Cas13a-based biosensor using combination of crRNAs. Two or three crRNAs targeting distinct regions of SARS-CoV-2 genome are mixed to increase the sensitivity of Cas13a-based detection. **(B)** Schematic of ultralocalized Cas13a assay. The Cas13a reaction is confined in cell-like-sized reactor to enhance the local reaction concentration. **(C)** Schematic of opn-SATORI. In this detection, biotin-labeled Cas13a complexes are captured by streptavidin-labeled magnetic beads, which are concentrated by the magnetic force into microchambers and are activated in the presence of target RNAs. **(D)** Schematic of casCRISPR. The trans-cleavage activities of both Cas14a and Cas13a reactions are orderly combined to detect targets.

The outbreak of coronavirus disease 2019 (COVID-19) caused by severe acute respiratory syndrome coronavirus 2 (SARS-CoV-2) has led to millions of deaths worldwide. Although most patients experience minor symptoms, people with low-level infections, especially asymptomatic carriers, may accelerate the spread of the virus; thus, an applicable detection approach is required for timely and accurate detection of SARS-CoV-2 (Bai et al., 2020). RPA-SHERLOCK has been utilized to detect SARS-CoV-2 by fluorescence and lateral-flow readout, with an LOD of 42 copies per reaction by targeting the S gene of SARS-CoV-2. Compared to RT-qPCR, the RPA-SHERLOCK assay achieved a sensitivity and specificity of 96 and 100%, respectively, using fluorescence readout, and 88% and 100%, respectively, using lateral-flow readout for detecting the S gene of SARS-CoV-2 in patient samples. In addition, the RPA-SHERLOCK method was optimized by adding an RNase contamination detection method on the lateral-flow test strip, thereby avoiding false positives or negatives resulting from exogenous RNase contamination (Patchsung et al., 2020). Furthermore, Zhang et al. developed a refined Cas13a-based test strip assay combining quantum dot microspheres with a Cas13a-based test strip for SARS-CoV-2 detection. In this assay, an LOD of 1 copy/ml was achieved in 1 h, with 100% specificity and 100% sensitivity compared to RT-PCR (Zhang Q. et al., 2022). Recently, SHERLOCK coupled with reverse transcriptase loop-mediated amplification (RT-LAMP) was used to detect 60 clinical samples of SARS-CoV-2, with 100% detection accuracy (Khan et al., 2021). Furthermore, several studies for extraction-free Cas13a detection of SARS-CoV-2 have also been reported. One study employed SHERLOCK and HUDSON integration to navigate epidemics (SHINE; the previously reported HUDSON-SHERLOCK method) for SARS-CoV-2 detection without RNA extraction; they also integrated RPA and the T7 transcription steps of SHERLOCK into one step to simplify the operating process and deployed an in-tube fluorescent readout, which could reduce the risk of contamination. The LOD of SHINE was as low as 10 copies/μl by testing HUDSON-treated saliva with an in-tube fluorescent readout in 50 min (Arizti-Sanz et al., 2020). Azmi et al. developed Cas13 assisted saliva-based and smartphone-integrated testing (CASSPIT), which further optimizes the HUDSON-SHERLOCK method by refining the chemical treatment and heat inactivation processes to detect saliva samples of SARS-CoV-2 with lateral-flow readout; the method achieved an LOD of 200 copies per reaction (Azmi et al., 2021). Notably, a recent study reported the RT-LAMP-CRISPR-Cas13a method based on SHERLOCK. This novel RNA extraction-free assay combined RT-LAMP, Cas13a reaction, proteinase K-heat inactivation (PK-HID), and exhibited 83% sensitivity and 100% specificity for the detection of SARS-CoV-2 in the nasopharyngeal samples compared to RT-qPCR. Meanwhile, positive predictive value (PPV) of 100% and negative predictive values (NPVs) of 81% for cycle threshold (CT) values of <20 were also verified using this method (Ortiz-Cartagena et al., 2022). Moreover, nucleic acid amplification-free methods for Cas13a-based detection of SARS-CoV-2 have made significant progress. A previous study reported a novel nucleic acid amplification-free method in which two or three crRNAs targeting distinct regions of the SARS-CoV-2 genome were combined to significantly increase the sensitivity of Cas13a-based detection (Figure 4A). This assay could achieve an LOD of 100 copies/μl within 30 min; furthermore, triple combinations of crRNAs enabled sensitive detection of as low as 31 copies/μl (Fozouni et al., 2021). Tian et al. recently established a neoteric method for nucleic acid amplification-free detection, called the ultralocalized Cas13a assay, which harnesses the bioinspired confinement effect by separating the reaction mixture of Cas13a, crRNA, target RNA, and RNA reporters into tens of thousands of monodisperse picoliter-sized droplets. This assay greatly increased the local concentration of the reaction and subsequently caused a more than 10,000-fold enhancement in sensitivity without nucleic acid amplification (Figure 4B). Using this method, they could detect as low as 6 copies/μl of the SARS-CoV-2 N gene, and absolute digital quantification was achieved (Tian et al., 2021). Furthermore, a novel nucleic acid amplification-free platform was developed recently, termed the automated platform on CRISPR-based nucleic acid amplification-free digital RNA detection (opn-SATORI), which combines the Cas13a reaction, microchamber technology, and magnetic beads technology. In this method, biotin-labeled Cas13a complexes are captured by streptavidin-labeled magnetic beads, which are concentrated by magnetic force into microchambers and are activated in the presence of target RNAs (Figure 4C). Using this assay, the authors detected as low as 3.9 copies/μl of SARS-CoV-2 RNA within 9 min and distinguished three variants of SARS-CoV-2: alpha, delta, and omicron (Shinoda et al., 2022). Furthermore, Heo et al. (2022) developed an electrochemical biosensor in which activated Cas13a cleaves the report RNA on the surface of the electrode to release methylene blue, resulting in an altered peak current. This method achieved an LOD of 4.4 × 10^−2^ and 8.1 × 10^−2^ fg/ml for the ORF and S genes of SARS-CoV-2, respectively. Recently, a Cas13a-based biosensor was established that employs the Cas13a reaction and graphene field-effect transistor (gFET) to test SARS-CoV-2 and respiratory syncytial virus, with an LOD of 1 aM. In this assay, activated Cas13a cleaved PolyU20 on gFET, leading to reduced electron transfer (Li et al., 2022b). In addition, a novel detection assay called CRISPR/CAS-based colorimetric nucleic acid detection (CASCADE) was reported, which combines the Cas13a system with gold nanoparticles (AuNPs) to allow naked-eye detection of SARS-CoV-2. LODs of 3 fM and 40 aM were obtained when the method was coupled with RPA and nucleic acid sequence-based amplification (NASBA), respectively. (López-Valls et al., 2022). PCR technology was also integrated with the Cas13a reaction: Niu et al. (2022) developed a PCR-CRISPR method to detect the HV69-70del mutant of SARS-CoV-2, with an LOD of 1 copy/μl, and utilized this approach to successfully distinguish various mutants of SARS-CoV-2. To avoid false replication in the reverse transcription process, a light-up CRISPR-Cas13 transcription amplification method was reported, in which transcription amplification without the reverse transcription process is initiated after specific recognition and ligation of the probe pairs, presubstrate A and presubstrate B; then, the specifically activated Cas13a digests an RNA aptamer, leading to a reduction in the fluorescence signal produced by the RNA aptamer-DFHBI-1 T complex. They detected MERS, SARS, SARS-CoV-2, and influenza viruses using this light-up detection method and identified a gene mutation in SARS-CoV-2 variants. Notably, the dual amplification and recognition of the light-up CRISPR-Cas13 transcription amplification method ensures its high sensitivity and specificity of detection (Wang Y. et al., 2021). Furthermore, Wang et al. (2022) recently established a CRISPR-Cas13a cascade-based viral RNA (Cas13C) assay, in which activated Cas13a cleaves the pre-primer into mature primer to promote the downstream transcription amplification to produce light-up RNA aptamers for generating fluorescence. Using this assay, they detected SARS-CoV-2 with an LOD of 0.216 fM per reaction and distinguished SARS-CoV-2 from its N501Y variant. In addition, a novel enzyme-free Cas13a-based detection technique coupled with hybridization chain reaction amplification, a type of toehold-mediated strand displacement, was employed to detect SARS-CoV-2; the method achieved LODs of 10 aM (6 copies/μl), 100 aM (60 copies/μl), and 10 aM (6 copies/μl) for the S, N, and Orf1ab genes of SARS-CoV-2, respectively, within 1 h (Yang et al., 2021).

### DNA viruses

SHERLOCK is not only for the detection of RNA viruses, but also confers the capability of Cas13a-based detection assay for diagnosis of DNA viruses through its RPA and T7 transcription processes. Several studies have verified the capacity of SHERLOCK for DNA virus detection (Khan et al., 2019; Wu et al., 2019; Wei et al., 2022). Canine parvovirus type 2 (CPV-2), a type of dog DNA virus, was detected by the RPA-SHERLOCK assay with an LOD of 100 amol/L of CPV-2 DNA within 30 min (Khan et al., 2019). Wu et al. (Wu et al., 2019) optimized RPA primers to make SHERLOCK work at 25°C and used it to detect DNA of the Epstein–Barr virus (EBV); this is the first report of room temperature detection using the SHERLOCK platform. The African swine fever virus (ASFV) was also detected by recombinase aided amplification SHERLOCK (RAA-SHERLOCK) with lateral-flow readout, achieving an LOD of 10 copies/μl within 1 h. This assay also exhibited significant specificity for ASFV with no cross-reactivity with other swine viruses and a 100% concordance rate with PCR results (Wei et al., 2022). Additionally, one research group established a rapid one-tube SHERLOCK detection system for ASFV by performing the RPA reaction and Cas13a reaction in one tube; the products of the RPA reaction in the inner tube enter the outer tube by centrifugation *via* two hydrophobic holes and then contact the Cas13a reaction system. This method could accomplish rapid virus detection within 25 min, with an LOD of 3 copies/μl (Hu et al., 2022). Besides, Cui et al. (2022) performed a picoinjection-aided digital reaction unlocking (PADLOCK) assay, which combines a droplet generator with the addition of the reaction initiator MgOAc through a droplet microfluidic device to avoid premature amplification, allowing detection of HPV16 at as low as 5 copies/μl. Recently, Zhang X. et al. (2022) established an RCA-PCR-CRISPR assay that combines the Cas13a reaction with dual amplification—rolling circle amplification (RCA) and PCR—to detect covalently closed circular DNA (cccDNA) of the hepatitis B virus (HBV). Using this approach, they could detect 1 copy/μL HBV cccDNA, with a higher positive detection rate than those of real-time PCR (qPCR), droplet digital PCR (ddPCR), RCA-qPCR, and PCR-CRISPR.

## Cas13a-based detection of bacteria and other pathogens

Food contamination and some severe diseases caused by bacteria and their toxins have led to an enormous burden on public health (Martinovic et al., 2016). Therefore, there is an urgent need to apply the appropriate methods for the detection of bacteria, especially in food safety inspection and infectious disease diagnosis (Zhou J. et al., 2020). The CRISPR/Cas13a system, as a sensitive, rapid, and specific technique, is being increasingly applied for the detection of bacteria. Some studies have focused on pathogenic bacteria of foodborne diseases. An et al. (2021) established a one-tube SHERLOCK method in which the RPA reaction and Cas13a reaction are performed in combination in a single tube and achieved detection of *Salmonella* spp. at as low as 10^2^ copies/μl within 20 min. Shen et al. (2020) developed a novel allosteric probe-initiated catalysis and CRISPR/Cas13a (APC-Cas) method to diagnose *S. enteritidis*. In the method, the presence of the target pathogen activates the allosteric probe-initiated amplification, and then, the ssRNA products promote crRNA-guided Cas13a collateral cleavage of the reporter probes to produce fluorescence signals. The APC-Cas assay could detect as low as 1 CFU/ml of *S. enteritidis* and exhibited superior specificity because of the dual recognition and triple amplification processes. Moreover, detection of viable bacteria is essential for bacterial virulence; therefore, one study developed a CRISPR/Cas13-based NASBA assay (called the cNASBA assay) that couples NASBA with the Cas13a reaction to detect and quantify viable *S. enterica* within 2.5 h, with an LOD of 1.5 CFU/ml of viable bacteria (Xue et al., 2022). In addition, Cas13a-based detection combined with PCR amplification was performed to detect *S. aureus*, another pathogen of foodborne diseases, exhibiting an LOD of 1 CFU/ml within 4 h (Zhou J. et al., 2020). In concordance with the detection of *S. aureus*, another study applied this PCR-Cas13a-based technique to detect *S. typhimurium* and achieved an LOD of as low as 1 CFU/ml (Gao et al., 2021). Furthermore, Zhang et al. (2021) deployed a Cas13a-based biosensor integrated with the light-up RNA aptamer technique to detect as low as 10 CFU/ml of *B. cereus*. They also confirmed the ability of this method for estimating the trend of food spoilage by quantifying the percentage of viable pathogenic bacteria. Diagnosis of infectious diseases caused by bacteria is also necessary. The ultralocalized Cas13a assay mentioned above was used to detect *P. aeruginosa* with an LOD of as low as 6 × 10^3^ copies/μl (Tian et al., 2021). Additionally, *Yersinia pestis*, the main pathogen causing plague, was detected by the RPA-SHERLOCK method with an LOD of as low as 700 zM (420 copies/ml; Schultzhaus et al., 2021). Using the RPA-SHERLOCK assay, Luo et al. (2021) detected 10 copies/μl of the porA gene of *Neisseria gonorrhoeae* and successfully identified two azithromycin resistance mutations.

In addition to detecting viruses and bacteria, the CRISPR/Cas13a system was also used for the detection of parasites, chlamydia, and fungus. Cunningham et al. (2021) employed RPA-SHERLOCK to achieve attomolar sensitivity for *Plasmodium falciparum* detection and achieved a desirable specificity for diverse *plasmodium* species. The Cas13a reaction coupled with RAA was also introduced to detect as low as 1 × 10^−6^ ng/μl of *Toxoplasma gondii* (Zhao et al., 2022). Additionally, a one-pot CRISPR/Cas13a assay was developed to detect dsDNA of *Chlamydia trachomatis* with an LOD of 10 fM (Huang et al., 2021). Recently, RPA-SHERLOCK was used to detect *Aspergillus fumigatus*, with an LOD of 3 copies/L within a total time of 70 min (Li et al., 2021). In addition, Zhan et al. combined transcription-mediated amplification (TMA) with Cas13a detection system and used this method to detect mitochondrial large subunit ribosomal RNA of Pneumocystis jirovecii, with an LOD of 2 copies/μl (Zhan et al., 2022).

Antimicrobial resistance (AMR) of pathogens has become a growing global public health concern during the past decade (Dean et al., 2022). The social and financial burden are estimated to be $100 trillion if this issue is not resolved (Piddock, 2016). Therefore, the detection of antimicrobial-resistant pathogens and subsequent rational drug use are vital for the containment of AMR. Cas13a-based detection methods could recognize SNP in target gene *via* artificially adding mismatch between crRNA and target RNA, exhibiting the potential to detect AMR mutants. Following to this mechanism, several studies have achieved detection of AMR mutants in different pathogens (Kiga et al., 2020; Cunningham et al., 2021; Luo et al., 2021; Bai et al., 2022). Cunningham et al. demonstrated the capability of RPA-SHERLOCK assay for recognizing the A581G mutant in *P. falciparum* associated with sulfadoxine resistance (Cunningham et al., 2021). Similarly, another group utilized RPA-SHERLOCK assay to identify the A2059G and C2611T mutants in Neisseria gonorrhoeae associated with azithromycin resistance, in accordance with sequencing (Luo et al., 2021). In addition, Bai et al. used Cas13a system with synthetic mismatch to significantly distinguish the wild type strains of Mycobacterium tuberculosis from fluroquinolone resistance mutant strains (G88A, A90V, S91P, D94N, D94H, D94T, and D94G; Bai et al., 2022). Moreover, another study established a novel method for detection of antimicrobial resistant Escherichia coli, termed CRISPR-Cas13a-based antibacterial nucleocapsids (CapsidCas13a). In this study, researchers constructed a bacteriophage capsid which contains programmed Cas13a system targeting AMR gene of Escherichia coli. Similar to drug sensitive test, Escherichia coli carrying AMR gene is unable to grow in the plate with the treatment of CapsidCas13a. Using this assay, they achieved detection of several AMR gene including blaIMP-1, blaOXA-48, blaNDM-1, and blaVIM-2 (Kiga et al., 2020).

## Cas13a-based detection of biomarkers

### Nucleic acid targets

Aberrant expression of microRNAs (miRNAs) is known to be associated with various tumors and has been confirmed as biomarker for initial cancer diagnosis (Chen et al., 2018). The detection of miRNA remains challenging given its low cellular abundance, small size, and high homology (Pritchard et al., 2012). Thus, the CRISPR/Cas13a system has been developed as a novel diagnostic tool to detect microRNA. Shan et al. (2019) first utilized the Cas13a system to directly detect as low as 4.5 amol of miR-17 and demonstrated its high specificity to distinguish SNPs in miRNAs. Subsequently, several studies refined the original CRISPR/Cas13a detection platform to detect miR-17. One study introduced a gold nanoparticle-based colorimetric assay integrated with the Cas13a system, allowing naked-eye detection of as low as 500 fM of miR-17 and promoting point-of-care detection based on the Cas13a assay (Yuan et al., 2020). Furthermore, a CRISPR/Cas13a-powered portable electrochemiluminescence chip, called the PECL-CRISPR, was established and could detect miR-17 at levels as low as 1 fM. In PECL-CRISPR, the activated Cas13a cleaves the pretrigger (PT) into mature trigger and then promotes downstream DNA amplification. Subsequently, the amplified dsDNA products interact with the light switch [Ru(phen)2dppz]2+ to produce the luminance signal. The PECL-CRISPR approach provided a single-nucleotide resolution for miR-17 together with its highly homologous family members and successfully analyzed the expression of miR-17 in human breast adenocarcinoma and human hepatocellular carcinoma cells (Zhou T. et al., 2020). Notably, instead of harnessing conventional methods for signal amplification, Sha et al. (2021) introduced a cascade CRISPR/Cas system (casCRISPR), which combines trans-cleavage activities of both Cas14a and Cas13a to detect miR-17 in an nucleic acid amplification-free manner (Figure 4D), with an LOD of 1.33 fM and high specificity for homologous miRNAs. As mentioned above, the ultralocalized Cas13a assay was also employed to detect and accurately quantify miRNA-17 in four different cell lines (Tian et al., 2021). In addition, other pivotal miRNAs have also been detected in previous studies. Bruch et al. exhibited a Cas13a-powered microfluidic electrochemical biosensor platform to quantify miR19b and miR20a, achieving an LOD of 10 pM within 4 h and confirming its feasibility for miR19b detection in serum samples of children suffering from medulloblastoma (Bruch et al., 2019). An electrochemical biosensor combining CHA with the Cas13a system was introduced to detect miR-21 and achieved an LOD of 2.6 fM using dual signal amplification of the Cas13a reaction and CHA amplification (Cui et al., 2021). Intriguingly, a novel magnetic relaxation switching (MRS)-based strategy was combined with the Cas13a system for the detection of as low as 0.22 pM of miR-21 with a unique T2 signal readout (Guo et al., 2022). Furthermore, Zhou et al. (2021) developed a Cas13a-powered rolling circle amplified DNAzyme colorimetric detection system, in which miRNA-activated Cas13a cleaves the pre-primer to initiate downstream DNA polymerase-mediated RCA. The resulting products catalyze the substrates to produce a color change. They could detect miR-10b with an LOD of as low as 1 fM using this method. In addition to miRNAs, lncRNAs are also considered significant biomarkers of cancers; thus, one group recently reported a plasmonically enhanced CRISPR-powered fluoroimmunoassay (p-FLISA), which integrates plasmonic-fluor as a fluorescent nanolabel with the Cas13a system. In this assay, p-FLISA was utilized to detect lncRNA H19 with an LOD of as low as 6 pM and could quantitatively detect H19 in human ovarian cancer cells and breast cancer cells. Furthermore, the feasibility of quantitative detection of tumor tissues obtained from a human ovarian cancer xenograft mouse model was confirmed (Liu L. et al., 2021). Recently, Song et al. (2022) reported a Cas13a-induced exponential amplification assay in which activated Cas13a cleaves stem-loop DNA primers to produce massive double stem-loop DNAs and promote loop-mediated isothermal amplification. Using this assay, they could detect circRNA sponge for miR-7 (ciRS-7) with an LOD of as low as 1 fM within 30 min and successfully discriminated circRNA from linear RNA. Notably, the diagnosis of clinical diseases by detecting a single specific biomarker is not specific; thus, it is indispensable to detect a set of biomarkers associated with a certain disease for accurate diagnosis. Bruch et al. (2021) refined a previously-developed Cas13a-powered microfluidic electrochemical biosensor platform into a multiplexed version, called CRISPR-Biosensor X, and realized synchronous quantification of a maximum of eight miRNAs. In another study, a Cas-CHDC-powered electrochemical RNA sensing technology (COMET) was developed, which combines the catalytic hairpin DNA circuit (CHDC) with the Cas13a system to detect and analyze six RNAs associated with non-small-cell lung carcinoma (NSCLC): miR-17, miR-155, miR-19b, miR-210, TTF-1 mRNA, and EGFR mRNA. This method could successfully discriminate between patients with early-stage NSCLC and patients with benign lung disease through combinatory analysis of multiple RNA biomarkers (Sheng et al., 2021).

### Non-nucleic acid targets

**Figure 5:**
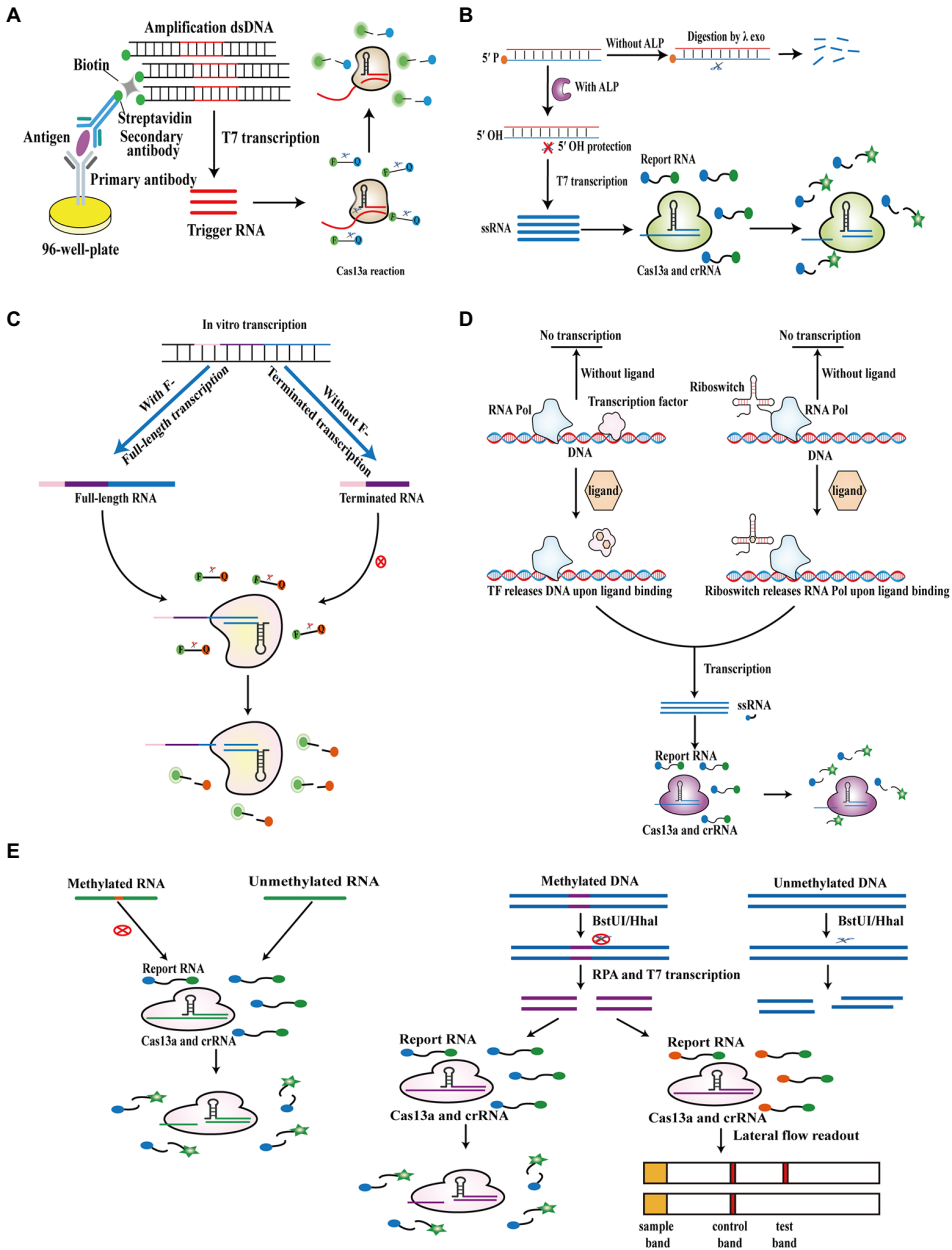
Cas13a-based biosensors for non-nucleic acid target detection. **(A)** Schematic of CLISA. Horseradish peroxidase in ELISA is replaced by biotinylated dsDNA containing a T7 promoter sequence to achieve Cas13a-based detection of antigen. **(B)** Schematic of TITAC-Cas. In the presence of ALP, 5′ P-dsDNA is converted into 5′ OH-dsDNA, avoiding digestion by λexo and activating the downstream T7 transcription and the Cas13a reaction. **(C)** Schematic of Cas13a-based biosensor for fluoride detection. In the absence of fluoride, template DNA is transcribed into terminated RNA, which cannot be recognized by the Cas13a system. Instead, in the presence of fluoride, template DNA is transcribed into full-length RNA, which can activate the downstream Cas13a reaction. **(D)** Schematic of SPRINT. The presence of ligands leads to the release of DNA by transcription factor or the release of RNA pol by riboswitch. Subsequently, the transcription products are recognized by the Cas13a system and activate the trans-cleavage activity of Cas13a. **(E)** Schematic of Cas13a-based biosensor for RNA and DNA methylation detection. Methylated RNA is not recognized by the Cas13a system due to the mismatch between methylated and unmethylated bases (above at left). Methylated DNA cannot be digested by BstUI/HhaI and then initiates the downstream RPA, T7 transcription, and the collateral cleavage activity of Cas13a (above at right).

Most researchers pay more attention to Cas13a-based detection of nucleic acids; however, the Cas13a system can also be employed for detecting non-nucleic acid targets. Chen et al. (2020) established a CRISPR/Cas13a signal amplification linked immunosorbent assay (CLISA), in which conventional horseradish peroxidase is replaced by dsDNA containing a T7 promoter sequence to activate the downstream Cas13a reaction system (Figure 5A). In this assay, two biomarkers, human IL-6 and VEGF, were detected with LODs of 2.29 fM and 0.81 fM, respectively. Wang et al. (2021a) developed a Cas13a-based detection assay that combines the target-induced transcription amplification with the trans-cleavage activity of Cas13a (called TITAC-Cas) and employs the dephosphorylation of alkaline phosphatase (ALP) as a switch to protect template dsDNA from digestion by λ exonuclease and activate the subsequent T7 transcription and trans-cleavage activity of Cas13a (Figure 5B). This assay allowed ultrasensitive detection of ALP activity (6 ± 0.52 mU/L). Another study established a Cas13a-based method for the detection of fluoride with an LOD of 1.7 μM, in which the presence of fluoride promotes the fluoride riboswitch to regulate transcription and produce full-length RNA, which then activates the collateral cleavage of downstream Cas13a to generate a fluorescence signal (Figure 5C; Ma et al., 2021). Furthermore, a high throughput analytical method called SHERLOCK-based profiling of *in vitro* transcription was reported, which combines ligand-dependent transcription activation of allosteric transcription factor and riboswitch with the Cas13a reaction. Because diverse ligands can be employed, it could quantify various compounds containing cofactors, nucleotides, metabolites of amino acids, tetracycline, and monatomic ions simultaneously based on ligand-dependent transcription (Iwasaki and Batey, 2020). Moreover, the Cas13a system was used to test the presence of methylation (Figure 5D). A study reported that the Cas13a system could be utilized to analyze methylation by artificially designed mismatches in crRNA based on the disability of methylated bases to pair with other bases. Based on this principle, two studies detected the presence of RNA methylation, including N1-methyladenosine (m1A) and N6-methyladenosine (m6A) and quantitated the percentage of methylated RNA in RNA samples (Figure 5E; Chen et al., 2019; Yang et al., 2020). Furthermore, Wang et al. (2021b) introduced a dual methylation-sensitive restriction endonuclease coupling with an RPA-assisted CRISPR/Cas13a System (DESCS), in which methylated DNA cannot be digested by two methylation-sensitive restriction endonucleases (BstUI/HhaI) and then initiates the downstream RPA, T7 transcription, and the collateral cleavage activity of Cas13a (Figure 5E). This approach could detect C5 methylation in the SEPT9 gene, with LODs of 86.4 aM and 200 aM using fluorescence and lateral-flow readout, respectively.

## Discussion and conclusion

So far, scientists have discovered two classes of the CRISPR-Cas system, among which class II effectors are expected to be widely employed because of their simplicity and high efficiency. Although some Cas9-based detection methods have been developed, the application of the CRISPR-Cas system as a biosensor only came under the spotlight when the collateral activity of Cas13a was reported. Currently, mainstream detection methods include PCR-based technologies, antigen–antibody reaction, genome sequencing, etc. (Yin et al., 2021). PCR-based methods are currently considered the gold standard of detection; however, PCR-based methods usually require expensive instruments, complete laboratory settings, and professionals, thereby limiting their deployment in low-resource regions, such as Africa (Kevadiya et al., 2021). Antigen–antibody reaction exists disadvantages of cross-reactivity and low sensitivity. Genome sequencing is time-consuming and highly expensive. Compared to these traditional diagnostic tools, CRISPR/Cas13a based detection methods are able to simultaneously achieve high sensitivity, high specificity, low cost and low time-consuming, by combining Cas13a system with isothermal amplification technique, visual readout, extraction-free method, etc. The SHERLOCK platform reported by Gootenberg et al. (2017) combined RPA nucleic acid amplification with the collateral activity of Cas13a; the dual signal amplification achieved by this method allowed attomolar sensitivity. Compared to RT-qPCR, the SHERLOCK assay is based on isothermal amplification (37–42°C) and does not require an expensive thermal cycler; furthermore, the SHERLOCK paper test just costs $0.61 per test. Subsequently, in SHERLOCKv2, an auxiliary enzyme was introduced, which significantly improved the detection sensitivity. Moreover, a lateral-flow strip readout was also added to SHERLOCK for point-of-care detection. Inspiringly, the SHERLOCK platform has gained approval for the detection of SARS-CoV-2 under emergencies by the Food and Drug Administration.

Since the SHERLOCK assay was developed, numerous studies have refined Cas13a-based biosensors by improving the SHERLOCK method or coupling the Cas13a reaction with advanced technologies. The multiple steps of SHERLOCK may cause aerosol pollution, operation complexity, and loss of sample; additionally, the amplification steps might be accompanied by amplification bias. Accordingly, a range of refined methods have been developed to simplify the complicated steps and reduce the testing time. Some studies utilized nucleic acid amplification-free methods, such as multiple combinations of crRNAs, ultralocalized Cas13a-based detection, opn-SATORI, and cascaded collateral activities of two Cas effectors (Fozouni et al., 2021; Sha et al., 2021; Tian et al., 2021; Shinoda et al., 2022). One extraction-free approach called HUDSON was also reported (Myhrvold et al., 2018). Furthermore, some studies combined the RPA and Cas13a reactions in a single tube, avoiding sample contamination during the transition of different steps and significantly reducing the total test time (Arizti-Sanz et al., 2020; An et al., 2021; Hu et al., 2022).

The programmability of crRNA makes the Cas13a system capable of detecting nearly all nucleic acid targets. Currently, Cas13a-based detection has been deployed to test pathogens, such as viruses, bacteria, parasites, chlamydia, and fungi; and biomarkers, such as microRNAs, lncRNAs, and circRNAs. The Cas13a system can effectively perform DNA detection because of transcription after amplification. Moreover, some non-nucleic acid targets, including proteins and ions, have also been detected by combining novel methods with the Cas13a reaction. Additionally, methylation and SNPs were successfully detected based on the mismatches between the guide and target. However, for the detection of methylation, the current methods can only test known methylation sites for supplementary validation. Furthermore, different readout methods, such as fluorescence readout, lateral-flow readout, colorimetric readout, and electrochemical readout, have been combined with the Cas13a reaction for detection.

The ideal detection approach is expected to be rapid, sensitive, specific, inexpensive, easy, and deployable. The majority of Cas13a-based biosensors still require multiple steps, including nucleic acid extraction, amplification, and the Cas13a reaction, which prolong the detection time. One-pot or tube methods shorten the detection time but they always give up high sensitivity. Combinations of crRNAs significantly increase the sensitivity of detection, whereas the capacity of this method to detect SNP remains to be verified (Fozouni et al., 2021). The sensitivity of a single Cas13a reaction is low, and nucleic acid amplification is still necessary to improve sensitivity. In addition, the issues of amplification bias and false-positive results caused by contamination during amplification remain to be solved (Yin et al., 2021; Li et al., 2022b). The prospective development of Cas13a-based detection is supposed to focus on improving the multiplexing capability by deploying a microfluidic system or through the orthogonal utilization of different Cas effectors. Ackerman et al., 2020 established a combinatorial arrayed reactions for multiplexed evaluation of nucleic acids (CARMEN) platform that can test more than 4,500 guide-target pairs on a single array and successfully detected 169 viruses. Since the casCRISPR system was developed, more different combinations of Cas proteins are expected to be further exploited. Furthermore, balancing sensitivity, availability, and operation time is also crucial for the future application of Cas13a-based biosensors, which remains to be studied further.

Effectively designing crRNA and clarifying the effect of the Cas13a system are vital for improving the detection efficiency. Tambe et al. (2018) demonstrated that there are two important functional regions in crRNA and target RNA: the seed region and the HEPN-nuclease switch region. Mismatches in the former lead to a significant reduction in the binding affinity, whereas mismatches in the latter inactivate the HEPN-nuclease without affecting the binding affinity. A previous study confirmed a 3′ protospacer flanking sequence called the anti-tag, which is complementary to the 5′ flanking region of the crRNA spacer, also termed as crRNA tag. This extra complementarity between the anti-tag and crRNA tag prevents the Cas13a-mediated cleavage of crRNA (Meeske and Marraffini, 2018). A subsequent structure analysis study demonstrated that the tag: anti-tag duplex results in conformational inactivation of the Cas13a protein (Wang B. et al., 2021). Intriguingly, (Li et al., 2020) found that targeting the NS3 region of DENV by the Cas13a system results in the deletion of the NS2B region, and the detection of NS3-crRNA alone without the Cas13a protein also provided an anti-viral effect. Thus, it is expected to clarify the specific effect mechanism of the Cas13a system to rationally select and design crRNAs and the Cas13a protein for accurate diagnosis.

In summary, the development and application of the CRISPR/Cas13a system may herald the emergence of a next-generation diagnostic tool, which will provide novel insights for the detection of diseases. Despite many advantages, there are still some challenges to be resolved. The ideal future development of Cas13a-based biosensors includes: (1) Improving the sensitivity of detection without relying on nucleic acid amplification methods (such as combinations of different Cas proteins, combinations of crRNAs, etc). The high-fidelity of Cas13a protein is often ignored as the efficient nuclease to recognize the amplification products (Zhou T. et al., 2020). (2) Combining sample treatment, amplification and Cas13a reaction into a real one-pot reaction that does not need additional manual operation and requires less time. (Soh et al., 2022). (3) Combining multiplexing technique with variable programmed ability of Cas13a system to achieve high throughput detection of various targets Ackerman et al. (2020). (4) Combining visual readout with mobile device to reduce artificial bias (Tian et al., 2022). Nevertheless, it is conceivable that the CRISPR/Cas13a system will become a promising diagnostic tool in future.

## Author contributions

LZ was involved in conceptualization, writing original draft, writing—review and editing, and visualization. MQ was involved in conceptualization and writing—review and editing. XL and JY were involved in writing—review and editing. JL was involved in conceptualization, writing—review and editing, and supervision. All authors contributed to the article and approved the submitted version.

## Funding

This work was supported by the National Natural Science Foundation of China (grant number 81570497) and the Science Foundation of AMU (grant number 21QNPY004).

## Conflict of interest

The authors declare that the research was conducted in the absence of any commercial or financial relationships that could be construed as a potential conflict of interest.

## Publisher’s note

All claims expressed in this article are solely those of the authors and do not necessarily represent those of their affiliated organizations, or those of the publisher, the editors and the reviewers. Any product that may be evaluated in this article, or claim that may be made by its manufacturer, is not guaranteed or endorsed by the publisher.
